# Validation and psychometric evaluation of physical activity belief scale among patients with type 2 diabetes mellitus: an application of health action process approach

**DOI:** 10.15171/hpp.2016.13

**Published:** 2016-06-11

**Authors:** Hosein Rohani, Ahmad Ali Eslami, Arsalan Ghaderi, Tohid Jafari-Koshki, Erfan Sadeghi, Mohammad Bidkhori, Mehdi Raei

**Affiliations:** ^1^Student Research Center, Faculty of Health, Isfahan University of Medical Sciences, Isfahan, Iran; ^2^Department of Health Education And Promotion, Faculty of Health, Isfahan University of Medical Sciences, Isfahan, Iran; ^3^Social Determinants of Health Research Center, Kurdistan University of Medical Sciences, Kurdistan, Iran; ^4^Department of Biostatistics, Faculty of Health, Tabriz University of Medical Sciences, Tabriz, Iran; ^5^Department of Biostatistics, Kermanshah University of Medical Sciences, Kermanshah, Iran; ^6^Department of Epidemiology, Sabzevar University of Medical Sciences, Sabzevar, Iran; ^7^Department of Basic Sciences, Qom University of Medical Sciences, Qom, Iran

**Keywords:** Health behavior, Psychometric properties, Physical activity, Diabetes mellitus, Validation, Health action process approach

## Abstract

**Background:** Moderate increase in physical activity (PA) may be helpful in preventing or postponing the complications of type 2 diabetes mellitus (T2DM). The aim of this study was to assess the psychometric properties of a health action process approach (HAPA)-based PA inventory among T2DM patients.

**Methods: ** In 2015, this cross-sectional study was carried out on 203 participants recruited by convenience sampling in Isfahan, Iran. Content and face validity was confirmed by a panel of experts. The comments noted by 9 outpatients on the inventory were also investigated. Then,the items were administered to 203 T2DM patients. Construct validity was conducted using exploratory and structural equation modeling confirmatory factor analyses. Reliability was also assessed with Cronbach alpha and interclass correlation coefficient (ICC).

**Results: ** Content validity was acceptable (CVR = 0.62, CVI = 0.89). Exploratory factor analysis extracted seven factors (risk- perception, action self-efficacy, outcome expectancies, maintenance self-efficacy, action and coping planning, behavioral intention, and recovery self-efficacy) explaining 82.23% of the variation. The HAPA had an acceptable fit to the observations (χ2 = 3.21, df = 3, P = 0.38; RMSEA = 0.06; AGFI = 0.90; PGFI = 0.12). The range of Cronbach alpha and ICC for the scales was about 0.63 to 0.97 and 0.862 to 0.988, respectively.

**Conclusion:** The findings of the present study provided an initial support for the reliability and validity of the HAPA-based PA inventory among patients with T2DM.

## Introduction

The incidence of type 2 diabetes mellitus (T2DM) is increasing worldwide.^1^ According to the recent studies in Iran, the prevalence of this disease is estimated to be 14.6% which introduce it as a leading cause of morbidity and mortality in the country.^2^ Seventy percent of the diabetic patients are living in low- and middle-income countries and the escalating burden of the disease is the highest in African and the Middle East countries.^3^ According to the estimates, up to 15% increase in the diabetes prevalence will be occurred in the developing countries, such as Iran, in the next 25 years.^4^

It is believed that T2DM complications can be prevented, or at least delayed, with a moderate increase in the physical activity (PA) and improvement in the diet of the patients.^5^ However, the most of T2DM patients in Iran do not participate in a regular, moderate-intense PA.^6,7^

An ongoing PA over time is a key factor to reap its benefits. However, it is shown to be difficult to design effective interventions to help patients in setting up and maintaining a healthy behavior like PA,^8,9^ especially among those with a sedentary lifestyle.^10^ As an approach to change behavior, it is presumed that if the cognitive beliefs associated with PA are known, more proper interventions may be developed to strength such beliefs.

**Figure 1:**
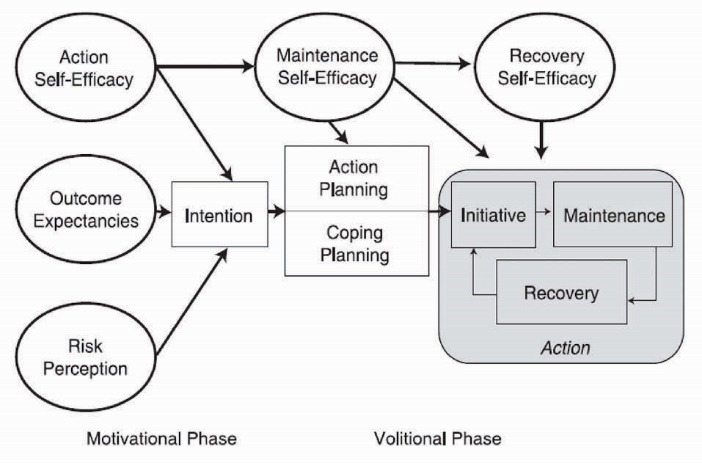
Diagram of the health action process approach (HAPA) model.

The hybrid model of health action process approach (HAPA) has been considered as a clearly specified identifier of the beliefs underling a wide range of health behaviors such as PA.^11^ This model may be conceptualized as a stage model, especially for the interventional purposes.^12^ Based on the HAPA (Figure 1), the process of health behavior change constitutes a sequence of motivational processes which results in intention. This is followed by volitional processes operating between intention and behavior enactment which help to fill in the gap between intention and behavior.^13^

There are distinct social cognitive predictors relevant and responsible for transition from each stage or mindset to the proceeding one. For non-intenders, like those at a non-intentional stage, a set of predictors such as risk perception, outcome expectancies and action self-efficacy that lead to form intention, may be the key targets for intervention.^14^ Risk perception in somebody refers to his/her understanding of being at risk for a certain health condition and may act as a trigger to start thinking about changing his/her health behavior. Outcome expectancies pertain to the expectations for positive rather than negative consequences that result from the behavioral change. Action self-efficacy is one’s belief about his ability to start the behavioral change. On the other hand, interventions with a target on the proximal predictors of behavior that mediate between intention and behavior are the most beneficial for intenders – those at the intentional stage. These constructs include action planning, coping planning, maintenance self-efficacy, and recovery self-efficacy.^15^ Action planning is to organize when, where, how and with whom one will do the intended behavior. Coping planning relates to the anticipations and strategies that somebody may take to overcome the hindrances that might obstruct the achievement of the intended changes. Maintenance self-efficacy and recovery self-efficacy are crucial for the initiation and maintenance of the behavioral changes. The first refers to possessing an optimistic belief about one’s ability to maintain the behavioral changes, and the latter refers to the confidence in his/her ability to resume the behavioral task after a holdup.^16^ In brief, the model suggests that there are theory-driven constructs which may determine the relevant targets of an intervention for people at the different stages of change.

To the best of our knowledge, there is no published instrument for measuring the HAPA-based beliefs on PA among T2DM patients. Therefore, in the present study, a set of scales was developed to measure the beliefs on PA applying HAPA model and also, the content and construct validity as well as internal consistency reliability and stability of the scales were investigated.

## Materials and Methods

### Participants and Procedures

**Figure 2:**
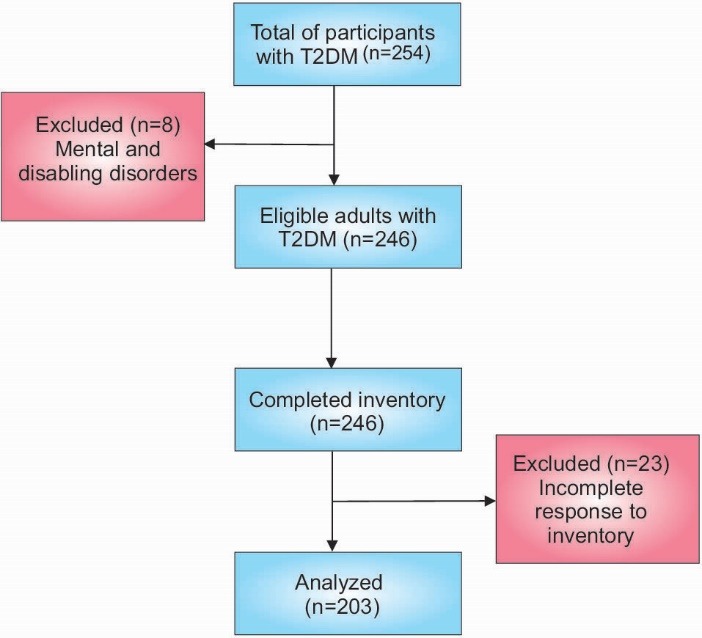
Study flow diagram for the participant recruitment.

To evaluate the HAPA-based beliefs scales among Iranian diabetic patients, a cross-sectional study was conducted on the participants recruited from Al-Zahra and Feyz hospitals, Isfahan, Iran from‏ January‏ to September 2015. The inclusion criteria for this study were being diagnosed with T2DM and having no history of T2DM complications. The exclusion criteria were mental and disabling disorders‏ indicated from their medical records and incomplete response to the inventory. Thus, the patients with diabetes complications were excluded from the study. The scale was re-administered to 20 individuals one month after the first visit for reliability assessment. The sample size of 5 to 10 patients per item in the model was considered as appropriate in order to run a structural equations modeling.^17^ Since there were 35 parameters in the model, 250 respondent seemed to be appropriate. The medical records of the patients were reviewed and, then, the qualified individuals based on the inclusion criteria were invited to participate in the study. At first, 254 patients were included in the study using convenience sampling and based on the inclusion criteria. Before each interview, the primary researcher briefly explained the purpose of the study to the participants. After applying the exclusion criteria, 8 patients were excluded and the rest of the patients filled in the inventory. The information of 23 patients was excluded from the data, because of incomplete responses (Figure 2). Finally, the subjects’ medical records were abstracted to be used as clinical and laboratory data.

### Translation

At first, the primary version of the scales was translated into Persian and then validated by forward-backward method.^18^ Also some phrases were replaced due to cultural differences. Finally, the validity and reliability of the translated version were evaluated.

### Face validity

To ensure the clarity and readability of the items, the HAPA inventory was administered to 9 T2DM patients (4 males and 5 females). Therefore, the items refinement‏ of the measures was conducted in terms of legibility and comprehensibility.

### Content validity

Five experts in the fields of biostatistics, health education and physical education were asked to rate, independently, on the necessity and relevance of the items in order to calculate content validity ratio (CVR) and content validity index (CVI), respectively. The necessity of the items (CVR) was evaluated using a three-point rating scale: (*i*) not necessary, (*ii*) useful, but not essential, (*iii*) essential. The relevance of the items (CVI) was also ascertained using a four-point rating scale: (*i*) not relevant, (*ii*) slightly relevant, (*iii*) relevant, and (*iv*) very relevant. The calculated CVI for each question was the proportion of the experts who voted for either third or fourth alternatives.^19^

### Construct validity

To assess the construct validity, inter-correlation and mutual exclusiveness of the items were explored using principal components analysis with a varimax rotation. Confirmatory factor analysis (CFA) was used to examine the measures as indicators for HAPA constructs.

It should be noted that the CFA was applied in another sample (n=201), which gathered via the same method and on the same target population, in order to prevent the overestimating of the model.

The confirmatory factor model determined the relationships between observed variables and unobserved latent variables.^20^

### Reliability

Cronbach alpha and its 95% of CI were calculated as an indication for internal reliability. Cut point of 0.70 was set for the Cronbach alpha to detect the acceptable items for the new scales. Temporal stability was assessed to determine the reliability over time. The questionnaire was completed by 20 patients after one month to calculate the test-retest reliabilities using the ICC.^21^

### HAPA inventory

The HAPA inventory comprises 8 scales; each item for a scale was rated on a seven-point scaling with anchors varying based on the content of the scales. All the scales aimed to performed PA as the outcome variable.

### Risk perceptions

Risk perceptions were obtained for each of the following five disorders: hypertension, high cholesterol levels, stroke, heart attack and cardiovascular disease. The participants’ perceived absolute own risk were assessed by asking questions about the likelihood of experiencing each of the aforementioned health problems. For example, “How high do you think is the risk of heart attack for you during your lifetime?” The participants rated their chances for developing the disorders in the future, using a separate seven-point scale ranging from 1=very unlikely to 7=very likely.^22^

The possible scores for this scale were ranged from 5 to 35, within which the higher scores indicates the higher perception from the risks of physical inactivity.

### Outcome expectancies

Patient expectancy was measured by nine items based on Ajzen’s recommendations.^23^ The participants were asked this question: “In your idea, what will be the consequences of engaging in PA for you, over the next two months?” Following this question, their responses were elicited to eight more specific‏ questions identified based on the previous research on the T2DM patients.^24,25^ A sample of the questions was : “If I stick to a moderate-intense PA, then…(*a*) it would be painful for me, (*b*) I would be better physically, (*c*) it would improve my body weight.” The participants expressed their agreement with the anchors of each pair using a seven-point scale ranging from 1=strongly disagree to 7=strongly agree. In this scale, the questions with opposite direction were scored, reversely. The possible score for this scale was ranged from 5 to 35, within which the higher score indicated the more positive outcome expectations of‏ doing PA.

### Action self-efficacy

Action self-efficacy was measured using a six-item scale based on Schwarzer^26^ guidelines for assessing task self-efficacy. The participants were asked to rate their confidence (from 1=not confident at all to 7=completely confident) in their physical ability to do, at least, a moderate-intense PA with no stop in one session of length 10, 20, 30, 40, 50, and 60 minutes if they were motivated enough to do so. The possible scores for this scale were ranged from 6 to 42. The higher scores indicated the better action self-efficacy toward PA.

### Behavioral intention

Behavioral intention was assessed with 3 items adapted from Ajzen:^27^ (1) “I intend to do at least 150 minutes per week moderate-intense PA in the next 2 months,” with responses from 1=extremely unlikely to 7=extremely likely; (2) “I will try to do at least 150 minutes per week moderate-intense PA in the next 2 months,” with responses from 1=definitely false to 7=definitely true; and (3) “I plan to do at least 150 minutes per week moderate-intense PA in the next 2 months,” with responses from 1=strongly disagree to 7=strongly agree. The possible scores for this scale were ranged from 3 to 21, within which the higher scores indicated the higher behavioral intention for doing PA.

### Action planning

To assess action‏ planning, we used‏ the four items‏ recommended by Schwarzer with a range from 1=strongly disagree to 7=strongly agree on whether‏ one‏ had‏ made‏ detailed‏ plans‏ with respect to his/her‏ PA‏ in‏ terms‏ of (*a*) how, (*b*) when, (*c*) where, and (*d*) with whom they will start PA.^26^ The possible score for this scale were ranged from 4 to 28, within which the higher scores indicated the more action planning for doing PA.

### Coping planning

In order to assess the coping planning, the same scale anchors as action planning with three items was used. The participants were asked to rate whether they had made detailed plans about (*a*) what to do if something interferes with PA; (*b*) what to do in tough conditions to stick to their intentions; and (*c*) when to especially watch out in order to stay committed. These items were based on the Schwarzer’s^13^ recommendations for assessing coping plans. The possible scores for this scale were ranged from 4 to 28, within which the higher scores indicated the more coping planning for doing PA.

### Maintenance self-efficacy

This scale was used to measure the participants’ confidence in their ability to do PA even if they had to overcome a certain barrier. According to the literature within the T2DM patients, nine barriers were identified.^28^ Tiredness, time limitation, lack of facilities, and bad weather conditions were examples of the barriers. Again, the similar seven-point scale was used to rate these items from 1=not confident at all to 7=completely confident. The possible scores for this scale were ranged from 9 to 63, within which the higher score indicated the higher maintenance self-efficacy toward PA.

### Recovery self-efficacy 

This scale measured‏ the participants’ opinions on getting back on track after relapse‏ or his/her capability to regain control after a failure or setback.^26^ The participants were asked about their confidence to return to PA after quitting the behavior for a period of time‏. After provision a question in this regard, the answer choices were presented for the following four specific questions: “I am sure I can continue PA if I… (*a*) had postpone in my plans for several times, (*b*) have not done PA for a week, (*c*) have not done PA for a month, (*d*) were not able to deal with myself for sometimes.” The rating was, again, based on a seven- point scale ranged from 1=not confident at all to 7=completely confident. The possible scores for this scale were ranged from 4 to 28, within which the higher scores indicated the higher recovery self-efficacy toward PA.

### Scoring

All the items of HAPA inventory were based on a Likert score ranging from 1 to 7. In each scale, the scores of the participants on all the items were summed to achieve the total score for the scale.

### Physical activity behavior

The short form version of the International Physical Activity Questionnaire (IPAQ) was used to measure the participants self-reported moderate-to-vigorous intensity PA behavior over the past 7-days.^29^ The validity of IPAQ as a measure of PA behavior in Iranian population has been previously established.^30^ In the present study, we chose to modify the IPAQ somehow to remove the usual walking effects from the improvements induced by the PA in moderate-intense PA.

### Data analysis

Descriptive statistics were obtained for all the variables. Missing data were imputed by the median score for each person on each scale. This attributes a value to the scale without introducing any bias at a lower score. Kolmogorov-Smirnov test was used to check for normality.^31^ Ceiling and floor effects for each item was then calculated based on the lowest and the highest scores.^32^ Exploratory factor analysis (EFA) using‏ principal component analysis method with varimax rotation along with CFA was used to evaluate the construct validity. We also used Kaiser-Meyer-Olkin (KMO) measure and Bartlett test to evaluate the sampling adequacy to conduct a satisfactory factor analysis. The best structure were considered to be the one with the eigenvalue greater than 1 and factor loading equal to or greater than 0.4.^33^

We used the following four criteria to determine the fit of the model: Chi-square, root mean square error of approximation (RMSEA), adjusted goodness-of-fit index (AGFI), and parsimony goodness-of-fit index (PGFI). The chi-square test compares the covariance matrix implied by the hypothesized model to the one obtained from observed variables in the population.^17^ Hence, if this test was non-significant then the fit will be acceptable. Generally, an AGFI equal to 1.0 and or above 0.90 indicates a perfect and an acceptable fit, respectively. Also, a model with a RMSEA value less than 0.08 is usually considered to be adequate. A model with small values of PGFI is parsimonious and fits the data well.^34^All the analyses were done using SPSS v. 18 and Amos v. 21.

## Results

The participants (n=203) were, mostly, male (56%) and married (80%). The mean age was 48.51 (SD:‏ 18.9) years and the educational status for the majority was high school diploma (31.5%). Further details are shown in Table 1.

**Table 1:** Summary of participant demographics (n = 203)

**Demographics**	**Number (%)**
Gender	
Male	118(58)
Female	85(42)
Education	
Not educated	48(23.4)
Elementary	31(15.4)
Secondary	36(17.5)
Diploma	64(31.5)
Graduated	19(9.5)
Postgraduate	5(2.7)
Marital status	
Single	27(13)
Married	161(80)
Divorced or widowed	15(7)
Income adequacy	
Adequate	16(8)
Not adequate	187(92)
	**Mean (SD)**
Age	48.51 (18.9)
Diabetes duration ‏(year)	11 (9.48)
Physical activity (minute)	109.01 (142.40)

### Face validity

Required changes to the original HAPA scales was minimal, and the most of changes were related to improving the visual arrangement of the inventory and also rephrasing questions in order to reduce complexity and ensure consistent comprehension.

### Content validity

According to Lawshe method, an item would be considered to have the minimum content validity if more than half of the panelists evaluated that item as essential.^35^ The calculated CVR for the total scale was 0.62 indicating a satisfactory result. Based on Polite and Beck recommendation, the values of 0.82 was considered as the acceptable lower limit for CVI.^36^ The agreement between the panelists was also found to be satisfactory (CVI=0.89).

### Construct validity

The result of the Kolmogorov–Smirnov test and Ceiling and Floor effects before performing factor analysis showed that there was no evidence against the normality (*P*>0.05).^37^Also less than 10% of the participants had the lowest and the highest possible scores (Table 2).

**Table 2:** Summary of HAPA inventory psychometric properties

**Scale**	**No. of items**	**Mean (SD)**	**Kurtosis**	**Skewness**	**Cronbach α**	**ICC**	**Floor effect (%)**	**Ceiling effect (%)**
Risk perception	5	22.38 (6.55)	1.20	-0.588	0.91	0.971	2.7	5.4
Action self-efficacy	3	13.83 (4.95)	1.69	-0.737	0.93	0.962	5.4	3.4
Outcome expectations	5	23.79 (5.86)	0.58	0.487	0.92	0.898	0	0
Behavioral intention	3	10.52 (4.73)	1.49	-0.162	0.63	0.862	6.1	1.4
Action and coping planning	7	25.06 (8.89)	1.55	-0.358	0.97	0.905	5.4	0.7
Maintenance self-efficacy	9	29.81 (10.05)	1.77	0.167	0.90	0.988	2.7	1.4
Recovery self-efficacy	4	15.6 (6.52)	1.34	-0.078	0.65	0.922	5.4	2.0

Factor structure was conducted using EFA. KMO statistic was 0.85 and the Bartlett test of sphericity was significant (*P*<0.0001). Seven factors were extracted as follows: Factor 1 was related to the maintenance self-efficacy items, with a range of 0.72 to 0.88 for factor loadings. Factor 2 included all the items from the action and the coping planning with a loading range from 0.66 to 0.76. The behavioral intention items fell into factor 3 with loadings from 0.69 to 0.79. Factor 4 represented the risk perception by factor loadings from 0.78 to 0.90. On the basis of the underlying constructs related to the items, the remaining three factors were named as follows: outcome expectancies, recovery self-efficacy, and action self-efficacy. The detailed results are shown in Table 3.

**Table 3:** Results obtained from exploratory factor analysis

**Construct**	**Items**	**F1**	**F2**	**F3**	**F4**	**F5**	**F6**	**F7**
Risk perception	High cholesterol level	-0.155	-0.004	-0.044	0.872^a^	0.054	0.1508	-0.009
	Heart attack	-0.080	-0.062	-0.093	0.894^a^	0.131	0.1405	0.006
	Hypertension	-0.063	-0.079	-0.023	0.903^a^	0.116	0.090	-0.008
	Osteoporosis	-0.066	0.019	-0.034	0.908^a^	0.088	0.120	-0.091
	Cardiovascular disease	0.138	-0.078	0.069	0.784^a^	0.127	0.091	0.073
Action self-efficacy	10 minute	0.205	0.183	0.236	0.057	0.147	0.041	0.899^a^
	20 minute	0.204	0.206	0.217	0.024	0.248	0.152	0.710^a^
	30 minute	0.210	0.196	0.364	-0.051	0.228	0.110	0.714^a^
Outcome expectancies	Improving weight	0.185	-0.029	0.375	-0.211	0.594^a^	0.151	0.426
	Improving blood sugar	0.025	0.023	0.130	0.116	0.826^a^	0.001	0.357
	Improving‏ blood cholesterol	0.086	0.067	0.011	0.111	0.910^a^	0.063	0.173
	I will be physically healthier	0.378	0.028	-0.106	0.154	0.807^a^	-0.089	-0.032
	I will be happier	0.264	-0.057	-0.099	0.220	0.863^a^	0.035	0.035
Behavioral intention	I intend to engage in physical activity over two next months	0.210	0.380	0.690^a^	0.098	-0.145	0.184	0.215
	I will try to engage in physical activity over two next months	0.249	0.316	0.756^a^	0.076	-0.055	0.145	0.246
	I will plan to engage in physical activity over two next months	0.247	0.269	0.796^a^	0.058	-0.039	0.112	0.188
Planning	When do physical activity	0.269	0.706^a^	0.363	-0.152	-0.122	0.033	0.326
	Where do physical activity	0.326	0.694^a^	0.439	-0.180	-0.085	0.018	0.259
	How do physical activity	0.320	0.668^a^	0.418	-0.130	0.074	-0.007	0.334
	With whom do physical activity	0.287	0.729^a^	0.382	-0.163	0.050	0.069	0.234
	What to do if something intervenes	0.286	0.766^a^	0.357	-0.170	0.042	0.084	0.229
	What to do in difficult situations in order to stick to my intentions	0.295	0.709^a^	0.359	-0.208	-0.003	0.034	0.193
	When to especially watch out in order to stay committed	0.312	0.691^a^	0.426	-0.137	0.118	0.066	0.133
Maintenance self-efficacy	It takes me long to make it a habit.	0.796^a^	0.280	0.189	-0.109	0.002	0.154	0.202
	Lack of facilities	0.849^a^	0.180	0.165	-0.047	0.057	0.100	0.235
	Time limitation	0.824^a^	0.261	0.140	-0.051	0.142	0.161	0.139
	Daily chores	0.859^a^	0.166	0.137	-0.017	0.174	0.079	0.161
	Lack of patience	0.881	0.158	0.121	-0.049	0.142	0.073	0.155
	Over weight	0.716^a^	0.475	.128	-0.024	0.079	0.222	-0.019
	Tiredness	0.868^a^	0.063	0.171	-0.047	0.201	0.078	0.141
	Not accompanied by family	0.718^a^	0.374	0.140	0.028	0.171	0.186	-0.066
	Bad weather conditions	0.722^a^	0.382	0.149	0.032	0.246	0.226	-0.115
Recovery self-efficacy	I postpone my plans several times	0.134	0.155	0.174	0.157	-0.106	0.864^a^	0.074
	I did not follow physical activity for a week	0.219	0.095	0.217	0.145	0.007	0.891^a^	0.059
	I did not follow physical activity for a month	0.171	0.101	0.220	0.216	0.127	0.891^a^	0.061
	I am not able to pull myself together sometimes	0.311	-0.046	0.257	0.254	0.082	0.801^a^	0.126
Eigen value		16.74	6.12	4.06	3.36	2.61	1.65	1.09
Explained variance (%)		37.93	14.04	9.15	7.72	6.08	3.64	2.34

Abbreviation: F, Factor. ^a^Most factor loading of each item among all factors.

All eigenvalues were greater than 1, and the total variance explained by the hypothesized model was 82.23%. In the exploratory stage, action planning and coping planning were merged as one construct named planning. Also three items of action self efficacy and two items of outcome expectancies were discarded because of not being loaded high enough on their related factors. Also, two items of the outcome expectancies scale were removed because of overlap and multiple factor loadings. The remaining items for the seven scales were kept for further analyses.

**Figure 3:**
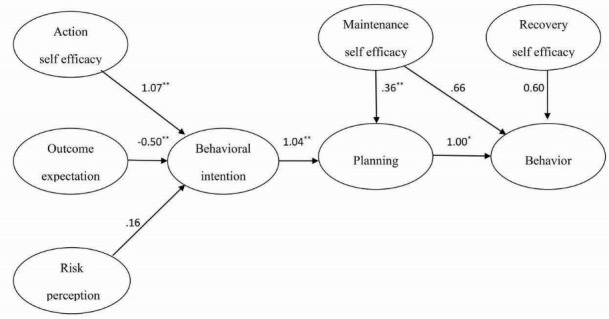
HAPA path model showing the significant standardized beta coefficients of the HAPA constructs. *P<0.01, **P<0.001.

CFA showed that all the scales were good indicators of their theoretical constructs. The fit to the PA measurement model was adequate (χ^2^=3.21, df=3, *P*=0.38; RMSEA=0.06; 90% CI=0.046-0.091; AGFI=0.90; PGFI=0.12). The path diagram of CFA is shown in Figure 3.

### Reliability

The summaries for internal consistency and test–retest reliability for each scale of the HAPA inventory are presented in Table 2. Most of the HAPA scales had good internal consistencies (α>0.90), and ICCs (ranged from 862 to 988).

## Discussion

In the current study, we evaluated a set of scales to measure HAPA concepts regarding PA among T2DM patients. Psychometric testing in this study provides preliminary evidence for validity and reliability of the HAPA-based questionnaire.

### Validity

Both factor analysis and structural equation modeling confirmed the construct validity of the scales. Nevertheless, further research is required to address some overlaps between the belief measures. It is evident that a person’s beliefs and cognitions may not occur independently and they may influence each other. Thus it may be impossible to sort out unique belief measures.^38^ The explained variance was relatively large which indicated that the PA is relatively well determined by HAPA. Our results are in accordance with the previous studies and confirm that HAPA constructs constitute the strongest predictors of intentions and PA.^11,14^

The results provided initial evidence for the validity of the HAPA-based measures. However, there may be a slight sample bias considering the nature of convenience sampling method, within which the collected data may not be representative for the target group. Also, external validity may be restricted.^39^

CFA has not been used in some of the recent validity studies^40,41^ which leads to an increased error in the analysis, since all the variables are not considered within a single model and therefore leads to a reduction in the power of the study.

Regarding this, it is highly recommended to use CFA while trying to construct a standard measure for health behavior studies.^42^ Accordingly, in the present study, we used structural equation modeling so as to confirm the hypothesized model which results in control for confounders’ bias reduction which increased the power of the study.

### Reliability 

Although, the Cronbach alpha was lower than 0.70 in some scales, the internal consistency of the HAPA inventory was acceptable. Also, no significant improvements were found in the Cronbach alphas after deletion of the related items. This may be a result of the low number of items included in the dimensions. Furthermore, small alpha coefficients may be due to small sample size, the high homogeneity of the patients, and the small variation of the scores.^43^ It seems that the reliability of the scales could be enhanced by increasing the sample size and the number of items in some dimensions as reported by Tan^44^ and Leung et al.^45^

### Generalizability 

Since there are various differences in health care services‏ provided for different communities (medical and non-medical),^46^ there seems to be a need for more investigation in order to figure out whether this questionnaire can be used in the other environments. For example, patients in hospitals are easily available and they mostly have enough time for answering the questions, a situation that may not be existed in the other places. On the other hand, in the present study, medical personnel were involved in data collection which may be resulted in more obedience among the patients to fill in the questionnaire.^47^

Because patients with progressed diabetes are mostly recommended with specific physical activities^48^ and our research was a prevention-based study, we considered not to include the patients with complications in the study. The reason for this exclusion was to introduce the obtained questionnaire as a scale to be utilized in a larger population who are at the early stages of the disease. Therefore, further considerations are necessary so as to fit the questionnaire in other groups of the diabetic patients such as patients with the progressed disease.

### Utilization

This study suggested scales that may be practically useful to assess the beliefs of diabetic patients in order to put more effective interventions into action. Valid and reliable scales to measure HAPA model beliefs give useful information on patients’ perception about those factors that facilitate or inhibit PA. This, in turn, may help in assisting the diabetic patients to improve their PA intentions and behaviors.^47^

### Limitations

There were two limitations for this study that restricted the interpretations of the results: the relatively small sample size and the small reliability values for some factors. Future studies using this questionnaire may settle these issues.

### Further research 

PA is a socially desirable behavior; hence, patients’ self-reported PA may be positively biased. It seems to be better to include a measure of social desirability, such as the Marlowe-Crowne Social Desirability Scale, in the future studies to evaluate this bias.^49^ Also, to check for the utilization of the presented questionnaire in the other environments and also the other groups of diabetic patients, additional researches are recommended.

## Conclusion

Although the results provided a preliminary support for validity and reliability of the HAPA inventory to examine the predictors of PA intentions and behavior among diabetic patients, it seems necessary to design more validation researches on this inventory applying an objective measure for PA to obtain additional support for its psychometric properties within the populations.

## Ethical approval

Isfahan University of Medical Sciences approved the study. At first, the participants were all informed that they could leave the study at any time they will. All the participants were assured about the anonymity of their information. At the end, the participants were rewarded with a credit card as a gift for their participation in the study.

## Competing interests

There is no conflict of interest.

## Acknowledgments

The authors would like to thank all staffs and patients for their participation in this study. This research was part of a PhD thesis of Isfahan University of medical sciences No: 394097. Research budget was paid by Isfahan University of medical sciences.

## Supplementary files

Supplementary file consists of

Appendix 1 Click here for additional data file..

## Authors’ contributions

HR and AAE contributed to the design of the work, performed data collection and analysis, drafted the manuscript, performed significant revisions, approved the final version of the manuscript. AG performed data collection and agreed to all aspects of the work. TF and ES Drafted the manuscript, performed significant revisions, approved the final version of the manuscript. MB and MR contributed to the design of the work, performed significant revisions and agreed to all aspects of the work.
