# Supplementary material for: Validation and psychometric evaluation of physical activity belief scale among patients with type 2 diabetes mellitus: an application of health action process approach
Source: Health Promot Perspect. 2016 Jun 11;6(2):71–9. doi: 10.15171/hpp.2016.13 (PMC4932225; doi:10.15171/hpp.2016.13)
Supplement: Appendix 1 [file hpp-6-71-s001.pdf]

Appendix

Health Action Process Approach Inventory

The following questions are about your physical activities. By physical activity, here, we mean 3 times a week activity, at least 30 minutes each, in which you can talk but cannot sing. Including brisk walking, running, biking, gardening and etc. Notice that routine walks as for doing daily tasks are not considered.

For each question, please put Crosshairs in each square that best describes your beliefs or feelings about being physically active. Please answer all of the questions.

Risk perception

A. How high do you think is your risk of following items during your life time?

1. High cholesterol level
2. Heart attack
3. Hypertension
4. Osteoporosis
5. Cardiovascular disease

Response rate:	very unlikely	1	2	3	4	5	6	7	Very likely

Action self-efficacy

B. Imagine you're highly motivated to do physical activities. How long are you sure you can do it within the next week?

1. 10 minutes
2. 20 minutes
3. 30 minutes

Response rate:	not confident at all	1	2	3	4	5	6	7	Completely confident

Outcome expectancies

C. What do you think will be the consequences for yourself if you engage in PA over the next two months?

1. Improving weight
2. Improving blood sugar
3. Improving blood cholesterol
4. I will be physically healthier
5. I will be happier

Response rate:	strongly disagree	1	2	3	4	5	6	7	strongly agree

Behavioral intention

D.

1. I intend to do at least 90 min per week moderate-intensity PA in the next 2 months.
2. I will try to do at least 90min per week moderate-intensity PA in the next 2 months.
3. I plan to do at least 90 min per week moderate-intensity PA in the next 2 months.

Response rate:	strongly disagree	1	2	3	4	5	6	7	strongly agree

Planning

E. How much do you agree/disagree with the following items?

1. I have planned when to do physical activity within the next week
2. I have planned where to do physical activity within the next week
3. I have planned how to do physical activity within the next week
4. I have planned with whom to do physical activity within the next week
5. I have planned what to do if something intervenes within the next week

6. I have planned what to do in difficult situations in order to stick to my intentions
7. I have planned especially when to watch out in order to stay committed

Response rate:	strongly disagree	1	2	3	4	5	6	7	strongly agree

Maintenance self-efficacy

F. Imagine you have started to exercise regularly. Considering the following obstacles, How sure are you to continue?

I am sure I can continue my physical activity 3 times a week regularly, at least 30 minutes each, even if…

1. It takes me long to make it a habit.
2. There is lack of facilities
3. There is time limitation
4. There are daily chores
5. There is lack of patience
6. I am over weight
7. I am tired
8. I am not accompanied by family
9. There are bad weather conditions

Response rate:	not confident at all	1	2	3	4	5	6	7	completely confident

Recovery self-efficacy

G. Imagine you have quit exercise. How sure are you to start it over again?

I can start doing exercises regularly even if…

1. I postpone my plans several times
2. I did not follow physical activity for a week
3. I did not follow physical activity for a month
4. I am not able to pull myself together sometimes

Response rate:	Not sure at all	1	2	3	4	5	6	7	Completely sure
